# Supplementary material for: Characterization of a Clinically and Biologically Defined Subgroup of Patients with Autism Spectrum Disorder and Identification of a Tailored Combination Treatment
Source: Biomedicines. 2024 Apr 30;12(5):991. doi: 10.3390/biomedicines12050991 (PMC11117897; doi:10.3390/biomedicines12050991)
Supplement: Supplementary file 1 [file biomedicines-12-00991-s001.zip › Supplementary Table S1.pdf]

Supplementary Table S1. Demographic data of ASD-Phenotype1, ASD-non-Phenotype1 and typically developing study participants

Participant	Gender	Head circumference (HC) percentile	Systematic aggravation of ASD behavioral symptoms (flares), during episodes of immune challenges such as fever and infection events	Age of participants at time of blood draw for LCLs (y.)	Biolog data (Y/N)	RNA profiling (Y/N)	Age of participants at time of blood draw for RNA-seq (y.)	Drug profiling in LCLs (Y(N)	ASD subgroups or TD
STPT000247	M	99	Improves with mild illness, worsens with severe, skin can be hot to touch but normal temp. Severe hives with illness. Mom states "massive insult with regression at 30 months and appeared to be related to illness".	10	Y	Y	10	Y	ASD-Phen1
STPT000248	M	>75	Behaviors worsen, speech regression with illness.	7	Y	Y	13	Y	ASD-Phen1
STPT000239	M	75	Worsens, does not want to talk, behaviors worsen.	27	Y	Y	27	N	ASD-Phen1

			Parents know he is sick due to these changes.						
STPT000298	M	90	Behavioral issues worsen significantly with illness. Regression at 15 months but cannot recall a specific illness.	2	Y	Y	4	N	ASD-Phen1
STPT000281	M	89	Behavior worsens drastically with illness and fever, agression, he is nonverbal, so speech is not affected.	2	Y	Y	6	N	ASD-Phen1
STPT000261	M	95	Behaviors worsen with illness, very irritable, less communication.	13	Y	Y	18	N	ASD-Phen1
STPT000289	M	>97	ASD symptoms worsen with illness, drastic change (parents can tell when he is getting sick by the shift in behaviors) less	7	Y	Y	10	N	ASD-Phen1

			communication when sick.						
STPT000274	M	90	ASD symptoms worsen with illness, he has confusion and fogginess, becomes more isolated. High fevers and facial flushing.	6	Y	Y	10	N	ASD-Phen1
STPT000258	M	89	Speech regression with illness (limited to sounds) "shuts down". School checks temps due to hot to the touch, but has normal temps. "red ears with illness"	3	Y	N	NA	N	ASD-Phen1
STPT000256	M	82	Worsens with illness and fever, no sounds, sensory overload, gets "real hot and flushed"	5	Y	N	NA	N	ASD-Phen1

STPT000257	M	84	Communication regression with illness. He is not sick often per mom.	6	Y	N	NA	N	ASD-Phen1
STPT000280	M	88	Hypersensitive, fixated, emotional disturbances when sick.	5	Y	N	NA	N	ASD-Phen1
STPT000263	M	95	Social intolerance during illness. At 2 years old had a significant viral illness that affected his hips. Was evaluated at MUSC where the MD indicated it was a virus that caused the orthopedic presentation. ASD dx wasn’t made until age 7.	6	Y	N	NA	N	ASD-Phen1
STPT000283	M	85	Symptoms worsen with illness, moody, frustrates easily, whiny.	9	Y	N	NA	N	ASD-Phen1

STPT000284	M	>95	Fatigued, irritable, less interactive, same applies when loss of a tooth.	4	Y	N	NA	N	ASD-Phen1
STPT000253	F	>97	Symptoms worsen with illness: aggressive, insomnia, fear and anxiety, inability to focus, less communication when sick. Parents can tell when she is getting sick due to changes in behavior.	1	Y	N	NA	N	ASD-Phen1
STPT000246	F	90-95	Symptoms worsen with illness. Irritability, hostility.	5	Y	N	NA	N	ASD-Phen1
STPT000254	M	>75	High temps when sick and sound sensitivity. He had several dental absesses and his symptoms worsened significantly.	16	N	Y	22	N	ASD-Phen1

STPT000266	M	> 97	Behaviors worsen, speech regression with illness. Rigid, controlling, and angry when sick.	15	N	Y	21	N	ASD-Phen1
STPT000260	M	90	Yes, at 12-24 months ran fevers of unknown origin as high as 103 and he was nonverbal at the time. He was diagnosed at 3 with ASD. He hasn't been sick since he was 24 months.	6	N	N	NA	N	ASD-Phen1
STPT000304	M	3	NA*	10	Y	Y	13	N	ASD-non-Phen1
STPT000315	M	3	NA*	8	Y	Y	10	N	ASD-non-Phen1
STPT000320	M	4	NA*	2	Y	Y	4	N	ASD-non-Phen1
STPT000288	M	53	NA*	6	Y	Y	9	N	ASD-non-Phen1
STPT000314	M	25	NA*	6	Y	Y	8	N	ASD-non-Phen1
STPT000317	M	40	NA*	2	Y	Y	4	N	ASD-non-Phen1
STPT000297	M	45	NA*	5	Y	Y	6	N	ASD-non-Phen1
STPT000291	M	67	NA*	3	Y	Y	5	N	ASD-non-Phen1
STPT000245	M	68	NA*	4	Y	Y	10	N	ASD-non-Phen1

STPT000240	F	3	NA*	19	Y	Y	21	N	ASD-non-Phen1
STPT000309	M	5	NA*	6	Y	N	NA	N	ASD-non-Phen1
STPT000244	M	20	NA*	7	Y	N	NA	N	ASD-non-Phen1
STPT000319	M	25	NA*	4	Y	N	NA	N	ASD-non-Phen1
STPT000241	M	30	NA*	6	Y	N	NA	N	ASD-non-Phen1
STPT000322	M	40	NA*	2	Y	N	NA	N	ASD-non-Phen1
STPT000318	M	<3%	NA*	4	Y	N	NA	N	ASD-non-Phen1
STPT000316	M	55	NA*	2	Y	N	NA	N	ASD-non-Phen1
STPT000324	M	57	NA*	5	Y	N	NA	N	ASD-non-Phen1
STPT000290	M	60	NA*	10	Y	N	NA	N	ASD-non-Phen1
STPT000285	F	NA	NA	3	Y	N	NA	N	TD
STPT000286	M	NA	NA	6	Y	N	NA	N	TD
STPT000292	F	NA	NA	7	Y	N	NA	N	TD
STPT000293	M	NA	NA	4	Y	N	NA	N	TD
STPT000294	M	NA	NA	4	Y	N	NA	N	TD
STPT000295	M	NA	NA	5	Y	N	NA	N	TD
STPT000296	M	NA	NA	5	Y	N	NA	N	TD
STPT000299	M	NA	NA	6	Y	N	NA	N	TD
STPT000300	M	NA	NA	5	Y	N	NA	N	TD
STPT000301	M	NA	NA	5	Y	N	NA	N	TD

STPT000302	M	NA	NA	7	Y	N	NA	N	TD
STPT000303	M	NA	NA	6	Y	N	NA	N	TD
STPT000305	F	NA	NA	6	Y	N	NA	N	TD
STPT000306	M	NA	NA	5	Y	N	NA	N	TD
STPT000307	F	NA	NA	3	Y	N	NA	N	TD
STPT000308	M	NA	NA	3	Y	N	NA	N	TD
STPT000310	M	NA	NA	7	Y	N	NA	N	TD
STPT000311	M	NA	NA	3	Y	N	NA	N	TD
STPT000312	F	NA	NA	8	Y	N	NA	N	TD
STPT000313	M	NA	NA	3	Y	N	NA	N	TD

*ASD-non-Phen1 patients were selected among patients with idiopathic ASD with a HC $<$ 75th percentile. No further assessments were made on these patients.
