# Supplementary material for: Characterization of a Clinically and Biologically Defined Subgroup of Patients with Autism Spectrum Disorder and Identification of a Tailored Combination Treatment
Source: Biomedicines. 2024 Apr 30;12(5):991. doi: 10.3390/biomedicines12050991 (PMC11117897; doi:10.3390/biomedicines12050991)
Supplement: Supplementary file 1 [file biomedicines-12-00991-s001.zip › Supplementary Table S2 .pdf]

Supplementary Table S2. Pathways significantly enriched for genes showing a statistically significant differential expression in ASD-Phen1 vs. ASD-non-Phen1

GeneSet_DB	Term	Overlap	*P*-value	Adjusted *P*-value	Odds Ratio	Genes
MsigDB_Hallmark_2020	TNF-alpha Signaling via NF-κB	2/200	0.004	0.017	24.99	*BCL2A1; PNRC1*
KEGG_2021_Human	Transcriptional Misregulation in Cancer	2/192	0.004	0.024	26.05	*BCL2A1; PAX5*
Reactome_2022	MyD88:MAL(TIRAP) Cascade Initiated On Plasma Membrane R-HSA-166058	2/112	0.001	0.026	45.18	*S100A12; S100A8*
Reactome_2022	Toll Like Receptor 4 (TLR4) Cascade R-HSA-166016	2/140	0.002	0.026	35.96	*S100A12; S100A8*
Reactome_2022	Diseases of Base Excision Repair R-HSA-9605308	1/5	0.002	0.026	555.17	*NEIL3*
Reactome_2022	Toll-like Receptor Cascades R-HSA-168898	2/162	0.003	0.026	30.98	*S100A12; S100A8*
Reactome_2022	RUNX1 Regulates Transcription of Genes Involved in BCR Signaling R-HSA-8939245	1/6	0.003	0.026	444.11	*PAX5*
Reactome_2022	Metal Sequestration by Antimicrobial Proteins R-HSA-6799990	1/6	0.003	0.026	444.11	*S100A8*
WikiPathway_2021_Human	ID Signaling Pathway WP53	1/16	0.008	0.028	147.96	*PAX5*
WikiPathway_2021_Human	Regulation of Apoptosis by Parathyroid Hormone-Related Protein WP3872	1/22	0.011	0.028	105.66	*BCL2A1*
WikiPathway_2021_Human	Base Excision Repair WP4752	1/31	0.015	0.028	73.93	*NEIL3*

WikiPathway_2021_Human	Prion disease pathway WP3995	1/33	0.016	0.028	69.30	*PAX5*
WikiPathway_2021_Human	Photodynamic Therapy-Induced NF-κB Survival Signaling WP3617	1/35	0.017	0.028	65.22	*BCL2A1*
WikiPathway_2021_Human	Photodynamic Therapy-Induced HIF-1 Survival Signaling WP3614	1/37	0.018	0.028	61.59	*BCL2A1*
Reactome_2022	Advanced Glycosylation Endproduct Receptor Signaling R-HSA-879415	1/13	0.006	0.045	184.98	*S100A12*
Reactome_2022	MyD88 Deficiency (TLR2/4) R-HSA-5602498	1/17	0.008	0.045	138.71	*S100A8*
Reactome_2022	Regulation Of TLR By Endogenous Ligand R-HSA-5686938	1/18	0.009	0.045	130.54	*S100A8*
Reactome_2022	IRAK4 Deficiency (TLR2/4) R-HSA-5603041	1/18	0.009	0.045	130.54	*S100A8*
Reactome_2022	Nuclear Events Stimulated By ALK Signaling In Cancer R-HSA-9725371	1/19	0.009	0.045	123.28	*BCL2A1*
KEGG_2021_Human	Base Excision Repair	1/33	0.016	0.049	69.30	*NEIL3*
Reactome_2022	RHO GTPases Activate NADPH Oxidases R-HSA-5668599	1/24	0.012	0.050	96.46	*S100A8*
Reactome_2022	TRAF6-Mediated NF-κB Activation R-HSA-933542	1/25	0.012	0.050	92.44	*S100A12*
