# Supplementary figures and images for: Characterization of a Clinically and Biologically Defined Subgroup of Patients with Autism Spectrum Disorder and Identification of a Tailored Combination Treatment
Source: Biomedicines. 2024 Apr 30;12(5):991. doi: 10.3390/biomedicines12050991 (PMC11117897; doi:10.3390/biomedicines12050991)

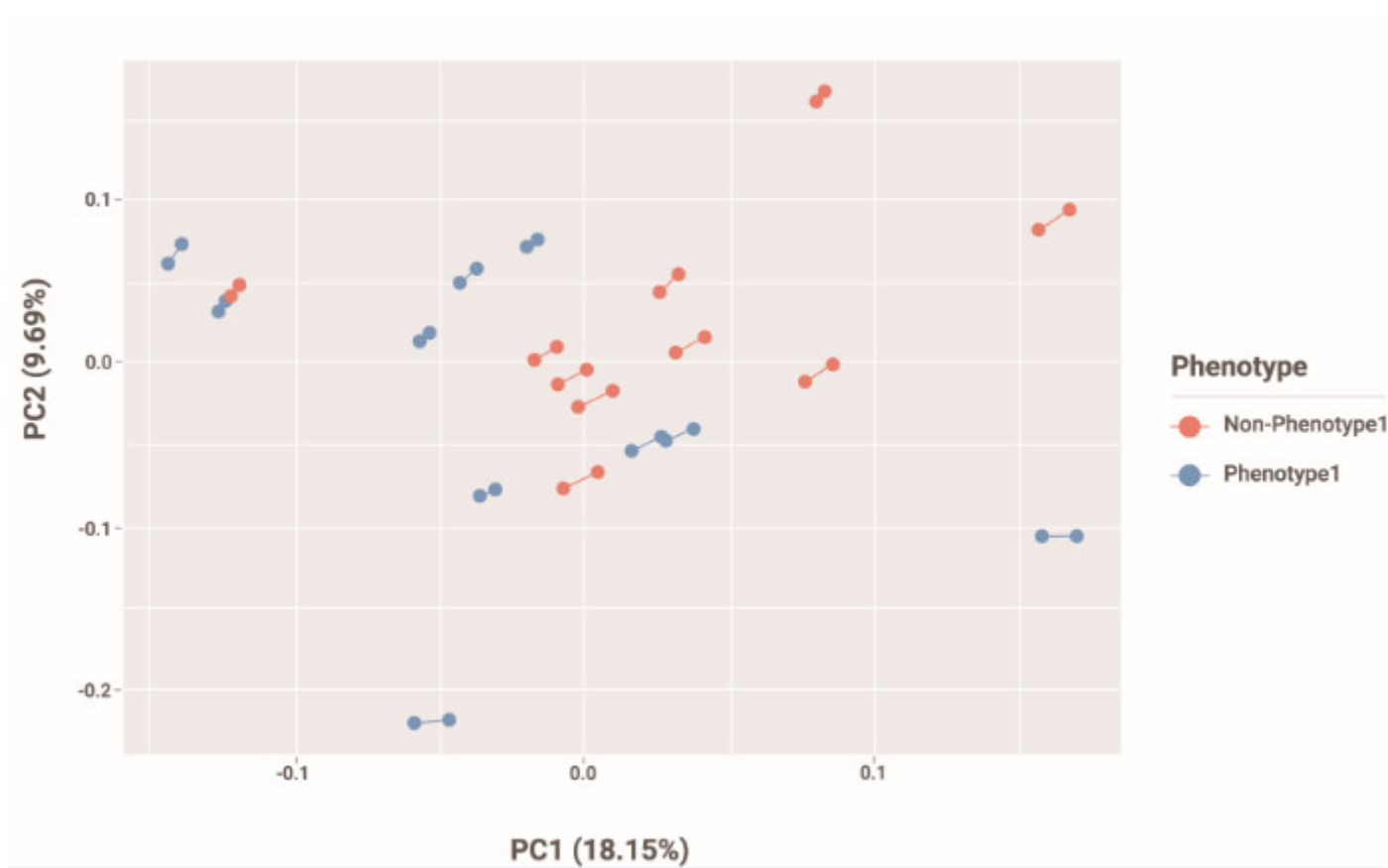

Figure S1: First two components of PCA using normalized gene expression values.

Supplement: Supplementary file 1 [file biomedicines-12-00991-s001.zip › Supplementary Figure S1.pdf]
